# Serum expression of selected miRNAs in patients with laryngeal squamous cell carcinoma (LSCC)

**DOI:** 10.1186/s13000-019-0823-3

**Published:** 2019-05-28

**Authors:** Weronika Lucas Grzelczyk, Janusz Szemraj, Sylwia Kwiatkowska, Magdalena Józefowicz-Korczyńska

**Affiliations:** 1 Department of Otolaryngology, Medical University of Lodz, Norbert Barlicki Memorial Teaching Hospital, Lodz, Poland; 2 Department of Medical Biochemistry, Medical University of Lodz, Lodz, Poland; 3 Department of Pneumonology, Norbert Barlicki Memorial Teaching Hospital, Lodz, Poland

**Keywords:** Circulating miRNAs, Laryngeal squamous cell carcinoma, Serum

## Abstract

**Background:**

The aim of the present study was to identify specific serum miRNAs (preoperative serum samples compared to healthy controls) as potential diagnostic markers for detection in laryngeal squamous cell carcinoma (LSCC). Serum samples obtained from 66 patients with LSCC were compared with 100 healthy control subjects. Additionally, miRNA levels were evaluated to identify possible correlations with clinicopathological features.

**Methods:**

The expression of 377 miRNAs (screening set) was evaluated by microarray screening. The most differentially expressed miRNAs were validated by high-throughput real-time quantitative polymerase chain reaction (RT-qPCR) in the group of LSCC patients and healthy controls. Receiver-operator characteristic (ROC) curve analysis was conducted to evaluate the diagnostic accuracy of the highly and significantly identified deregulated miRNA(s) as potential candidate biomarker(s).

**Results:**

According to the array analysis, eleven miRNAs revealed an altered expression profile. The levels of serum expression of miR-31, miR-141, miR-149a, miR-182, LET-7a, miR-4853p, miR-122 and miR-33 were up-regulated, and those of miR-145, miR-223 and miR-133a down-regulated, in the LSCC group compared to healthy controls. ROC curve analyses revealed an AUC (area under the ROC curve) of 1.00 (95%Cl: 0.999–1.00; *P* <  0.001) for miR-31 and LET-7a, 1.00 (95%Cl: 1.00–1.00; P <  0.001) for miR-33 respectively, indicating that these three miRNAs had an additive effect regarding diagnostic value. No statistically significant differences were found between the serum levels of these eleven miRNAs and the tested clinicopathological features.

**Conclusion:**

Our findings outline a distinct miRNA expression profile in laryngeal cancer (LC) cases which can be used to diagnose LSCC patients with high sensitivity and specificity. Particular miRNA signatures (miR-31, LET-7a and miR-33) may be considered as novel, non-invasive biomarkers for LC diagnosis.

**Trial registration:**

Registration number: RNN/203/13/KE. Date of registration 18.06.2013r.

## Background

Despite the great progress in chemotherapy and radiotherapy, as well as surgical techniques, laryngeal cancer is responsible for more than 80,000 death every year worldwide [1]. The most frequent histological type of LC, laryngeal squamous cell carcinoma (LSCC), accounts for approximately 90% of all malignant larynx tumor [2] and is more prevalent in men. The main reason for the very low five-year survival rate, which is less than 50% in advanced stages of the disease, is late diagnosis; in fact, the survival rate for laryngeal cancer has remained unchanged for the past 30 years [3]. The most significant risk factors are smoking, human papillomavirus (HPV) infection and alcohol consumption [4].

MicroRNAs (miRNAs) are non-coding, single stranded RNAs about 22–23 nucleotides in length. Above 30% of human messenger RNAs (mRNAs) are controlled via the action of miRNAs. They regulate post-transcriptional gene expression by influencing the 3′-UTR-binding proteins of the target mRNAs, resulting in the cleavage of mRNA strands or the inhibition of their translation [5].

Numerous studies have shown that a particular miRNA can bind to as many as 200 gene targets. These targets can vary in their function: they can involve transcription and secreted factors, receptors and transporters [6].

These small non–protein-coding RNAs have been found to play a significant regulatory role in biological and pathological processes [5–8]. The alterations can be induced by a variety of mechanisms involving amplifications, deletions or mutations, including those of miRNA loci, epigenetic silencing or the deregulation of transcription factors that target particular miRNAs. Malignant cells demonstrate dependence on the deregulation expression of miRNA genes; under certain circumstances, miRNAs may perform as either oncogenes or tumor suppressors [7].

A significant body of data has suggested that miRNAs are highly expressed in numerous human cancers and play critical roles in human oncogenesis and metastasis [7–9].

The last decade has witnessed considerable growth in the volume of miRNA studies, and the goal of working up miRNA targeted therapies or using miRNAs for prognostic and diagnostic biomarkers is becoming ever closer [8]. There is a hence a great need for research into the value of miRNAs as expected targets for therapeutic and diagnostic development against malignant tumors [9].

The aim of the present study was to compare specific miRNA expression profiles in preoperative serum LSCC patients vs those in healthy controls, to identify potential diagnostic markers for detecting LSCC, and to evaluate a possible correlation with clinicopathological features.

## Methods

### Characteristics of study groups

The study group was formed of 66 patients (eight women and 58 men) with laryngeal squamous cell carcinoma, aged between 48 and 82 years (mean 62 ± 8.0 years). All of the patients were treated surgically at the Department of Otolaryngology and Oncological Laryngology of the Medical University of Łódź in years 2013–2017; none of the patients received preoperative radiotherapy, chemotherapy or immunotherapy. The primary tumor size and stage were classified according to the Union for International Cancer Control (UICC) and the American Joint Committee on Cancer (AJCC).

**Fig. 1:**
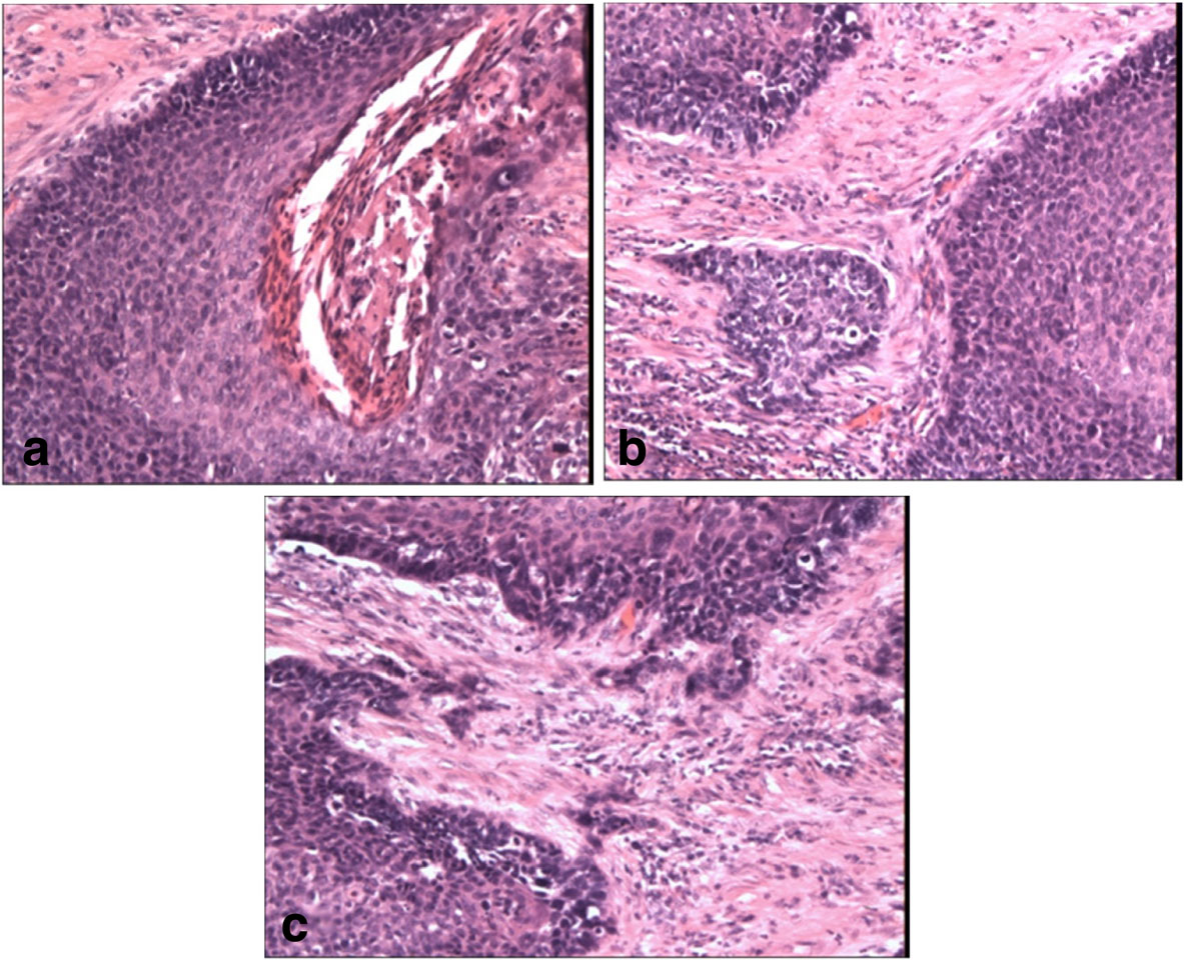
Histopathology of test specimens. **a** Well differentiated tumor tissues of LSCC (H&E, × 100). **b** Moderately differentiated tumor tissues of LSCC (H&E × 100). **c** Moderately differentiated tumor tissues of LSCC (H&E × 100). Granted permission Marian Danilewicz MD, PhD, Prof – Head of the Department of Pathomorphology, Medical University of Lodz, Poland

In order to evaluate the impact of the variables on the results of the miRNAs the patients were assessed with respect to sex (female, male), age (65 ≤ vs 65>), primary tumor size (T1, T2 vs. T3, T4), lymph node status (N0 –negative vs. N+ positive), histopathological staging (Fig. 1), (G1, G2, G3), clinical stage (I, II vs. III, IV), location of the carcinoma, and smoking duration (all the investigated LSCC patients were smokers from 20 to 100 pack-years, mean 44 ± 17 pack-years smoking). Clinical details are provided in Table 1.

**Table 1:** Characterization of the patients with laryngeal cancer (*N* = 66)

Feature	No. of patients (%)
Sex	
Female	8 (12.1)
Male	58 (87.9)
Age	
≤ 65	45 (68.2)
> 65	21 (31.8)
Primary tumor size (T)	
T1, T2	19 (28.8)
T3, T4	47 (71.2)
Nodal involvement (N)	
Negative	49 (74.2)
Positive	17 (25.8)
Histopathology grade	
Well differentiated	7 (10.6)
Moderately differentiated	53 (80.3)
Poorly differentiated	6 (9.1)
Clinical stage	
I, II	17 (25.8)
III, IV	49 (74.2)

In addition,, a control group composed of 100 healthy controls (nonsmokers with no history of cancer, hypertension, diabetes or autoimmune diseases) was formed. This group comprised 47 women and 53 men aged between 49 and 84 years (mean age 65.5 ± 7.5 years).

The study design was approved by the Ethics Committee (RNN/203/13/KE).

### Sample collection

Blood-serum samples were drawn from all the patients before the surgical treatment and from the 100 healthy controls. These were allowed to clot before centrifugation. The sera were removed, aliquoted and stored at −80 °C until assayed.

### RNA extraction and reverse transcriptase polymerase chain reaction

The miRNAs were isolated from 400 μl of serum and taken from laryngeal squamous cell carcinoma patients and controls using the mirVana PARIS Kit (Ambion) according to the manufacturer’s protocol [10]. The amount and quality of isolated RNA was checked with Agilent RNA 6000 Nano Kit, in accordance with the manufacturer’s recommendations, using 2100 Bioanalyzer (Agilent Technologies). Complementary DNA (cDNA) was transcribed from RNA using TaqMan®RNA Reverse Transcription kit (Applied Biosystems).

### Screening of laryngeal squamous cell carcinoma associated miRNA genes

Reverse transcription of isolated miRNA from ten laryngeal squamous cell carcinoma, patient blood samples and ten controls was performed according to the manufacturer’s recommendations using Megaplex™RT Primers Human Pool A and B, TaqMan® Human MicroRNA Array A and B purchased from Applied Biosystems using a 7900 HT System (Applied Biosystems). The expression levels of 377 human miRNA genes for each group were analysed. The following miRNAs revealed an altered expression profile: miR-122, miR-21, miR-155, miR-222, miR-181a, LET-7a, miR-31, miR-141, miR-149, miR-182, miR-145, miR-223, miR-133a, miR-485, miR-122, miR-33. These were chosen for further investigations.

### Methods of statistical analysis

Quantitative data (miRNA expression in serum) was described by simple descriptive statistics: mean, median values for location, standard deviation and range for dispersion.

The non-parametric Mann-Whitney test was used to compare their distributions in two independent groups: neoplastic and healthy control. The results were considered statistically significant for *p*-values ≤0.05.

The diagnostic performance of miRNAs in distinguishing between neoplastic and healthy control was evaluated with use of ROC (Receiver Operating Characteristics) curve analysis. Diagnostic efficacy for decision thresholds was evaluated with the use of chosen on ROC curve, was defined by sensitivity, specificity, and accuracy.

The area under the ROC curve (AUC) was used with a 95% confidence interval as an overall performance measure for distinguishing between the two diagnostic groups: the LSCC group and healthy controls.

All the calculations were performed using Statistica v12.0 software.

## Results

### Expression profiles of miRNAs in the serum of patients with LSCC

The panel of selected miRNAs was differently expressed (*p* <  0.05) in the sera of LSCC patients and healthy controls. The study was performed into two phases. The first evaluated the expression of 377 miRNAs using microarray screening (screening set) in the groups of LSCC patients *n* = 10 and healthy controls n = 10 to find out the most differentially expressed miRNAs.

Then eleven chosen miRNAs were validated in a different cohort of LSCC patients *n* = 66 and healthy controls *n* = 100 (validation phase) analyzed by RT-qPCR.

Validation set consisted of eleven miRNAs: eight were significantly up-regulated and three were significantly down-regulated.

**Fig. 2:**
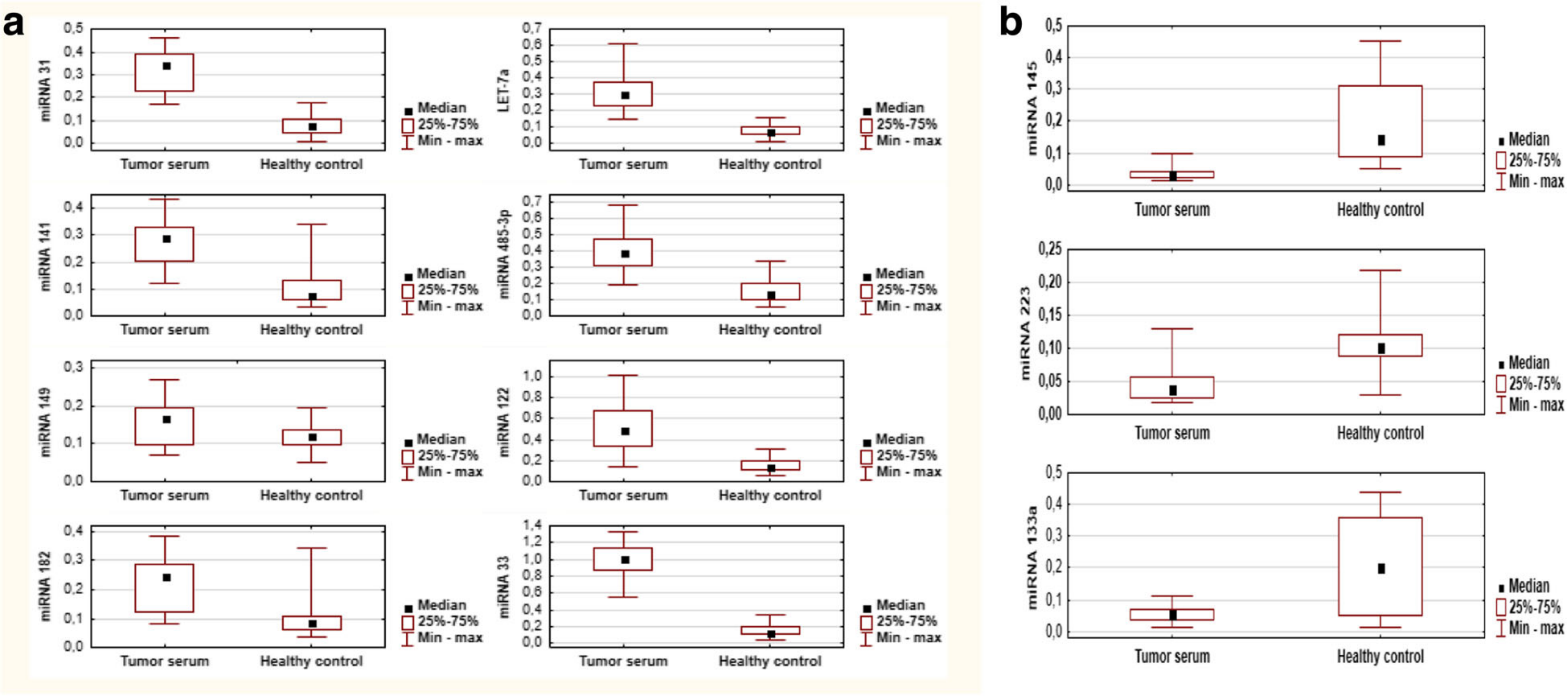
The qRT-PCR results of differential miRNAs. **a** Elevated expression of eight miRNAs in serum of LSCC patients compared with healthy individuals (healthy controls). The levels of miR-31, miR-141, miR-149a, miR182, LET-7a, miR-4853p, miR-122, miR-33 was measured in serum samples from LSCC (*n* = 66) and healthy control individuals (*n* = 100). **b** Down-regulated expression of miR-133a, miR223 and miR-145. Data represent the mean ± S.D

The expression of eight miRNAs miR-31, miR-141, miR-149a, LET-7a, miR-182, miR-4853p, miR-122, miR-33 was significantly up-regulated in the serum samples of the LSCC patients in comparison to healthy control samples (*p* <  0.001). The up-regulated miRNA expression levels are displayed in Fig. 2a. In contrast, the serum expression of miR-145, miR-223 and miR-133a was significantly down-regulated in the LSCC group compared to healthy controls (p <  0.001). The down-regulated miRNA expression levels in serum are displayed in Fig. 2b. The details are displayed in the Table 2.

**Table 2:** The results of eleven selected miRNAs analyzed by RT-qPCR

miRNA serum	Group	N	Mean+/−SD	*p*
miR-31	neoplastic	66	0,345 ± 0,090	< 0,001
	control	100	0,078 ± 0,036	
miR-141	neoplastic	66	0,289 ± 0,079	< 0,001
	control	100	0,077 ± 0,082	
miR-149a	neoplastic	66	0,168 ± 0,051	0,003
	control	100	0,120 ± 0,028	
miR-182	neoplastic	66	0,247 ± 0,091	< 0,001
	control	100	0,085 ± 0,076	
miR-145	neoplastic	66	0,031 ± 0,017	< 0,001
	control	100	0,146 ± 0,124	
miR-223	neoplastic	66	0,038 ± 0,032	< 0,001
	control	100	0,102 ± 0,035	
LET-7a	neoplastic	66	0,298 ± 0,110	< 0,001
	control	100	0,073 ± 0,034	
miR-133a	neoplastic	66	0,058 ± 0,024	0,007
	control	100	0,204 ± 0,156	
miR-485-3p	neoplastic	66	0,394 ± 0,111	< 0,001
	control	100	0,136 ± 0,078	
miR-122	neoplastic	66	0,497 ± 0,202	< 0,001
	control	100	0,147 ± 0,058	
miR-33	neoplastic	66	1013 ± 0,184	< 0,001
	control	100	0,136 ± 0,071	

Significantly differentially expressed miRNAs in the serum of LSCC patients (*n* = 66) when compared to healthy controls (*n* = 100). Data represent the mean ± S.D

**Fig. 3:**
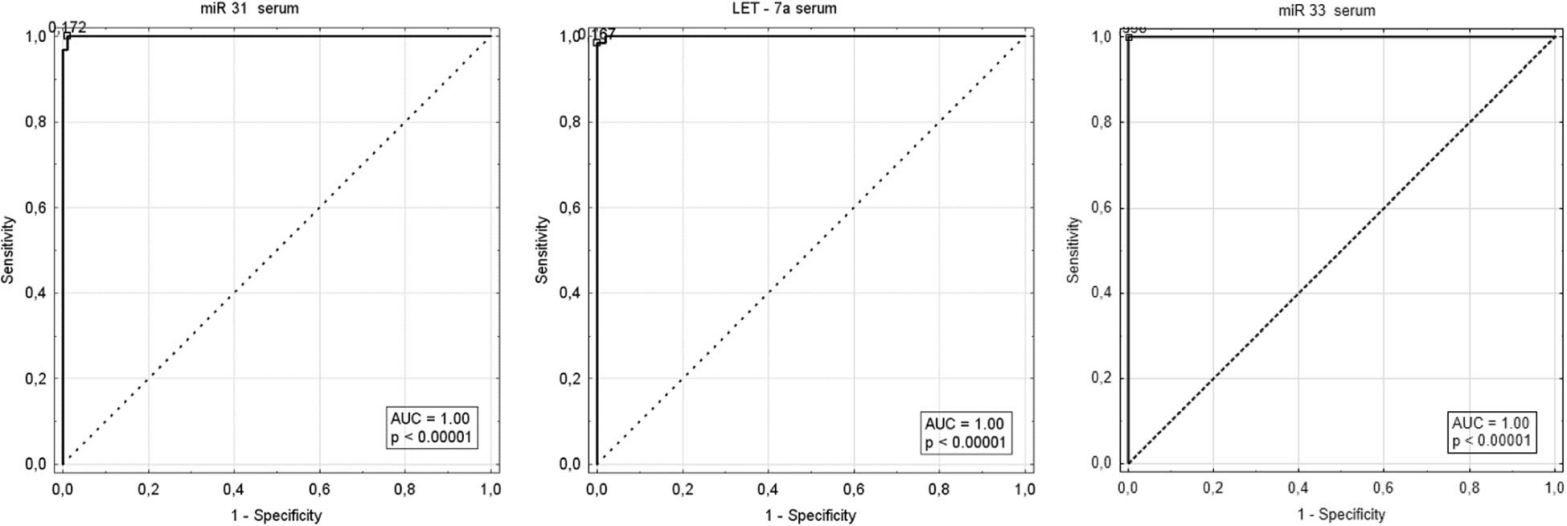
Receiver operator characteristic curve analysis using serum miR-31, LET-7a and miR-33 for discriminating LSCC patients. Serum miR-31, LET-7a and miR-33 were able to diagnose LSCC with a sensitivity of 100, 98, 100%, and specificity of 99,100 and 100%, respectively, as compared with healthy controls

In addition, ROC curve analyses indicated that three of the eleven serum miRNA signatures strongly differentiated healthy individuals and LSCC patients: miR-31, miR-33, Let-7a. The accuracy values were 99, 100 and 99%, respectively, with sensitivity and specificity ranging from 98 to 100%. The ROC curve analyses revealed that these three miRNAs could serve as valuable biomarkers for differentiating LSCC patients from controls. ROC curves were illustrated in Table 3, Fig. 3.

**Table 3:** The Receiver Operator Characteristic curve values for the expression of the eleven serum miRNAs and the corresponding calculated AUC

Serum miRNA	Threshold	AUC 95%Cl	*p*-value	Sensitivity [%]	Specificity [%]	Accuracy [%]
miR-31	**0.172**	**1.00 (0.999; 1.00)**	**< 0.000001**	**100**	**99**	**99**
miR-141	0.165	0.91 (0.87; 0.96)	< 0.000001	92	87	89
miR-149 a	0.154	0.68 (0.58; 0.77)	0.0005	58	91	80
miR-182	0.106	0.85 (0.79; 0.91)	< 0.000001	87	75	80
miR-145	0.052	0.98 (0.97; 1.00)	< 0.000001	94	99	97
miR-223	0.056	0.85 (0.79; 0.92)	< 0.000001	76	88	83
LET-7a	**0.167**	**1.00 (0.99; 1.00)**	**< 0.000001**	**98**	**100**	**99**
miR-133a	0.091	0.73 (0.65; 0.80)	< 0.000001	94	56	70
miR-485-3p	0.222	0.96 (0.94; 0.98)	< 0.000001	98	81	88
miR-122	0.232	0.96 (0.93; 0.99)	< 0.000001	89	94	92
miR-33	**0.558**	**1.00 (1.00; 1.00)**	**< 0.000001**	**100**	**100**	**100**

### Correlation of serum miRNA levels with clinical characteristics

The pretreatment serum expression levels of miR-31, miR-141, miR-149, miR-182, miR-145, miR-223, LET-7a, miR-133, miR-485-3p, miR-122, miR-33 in patients with LSCC were compared with the clinicopathological factors of the disease.

No statistically significant correlation was observed between serum levels of eleven miRNAs and the clinicopathological features of the disease concerning T stage, N stage, clinical stage or differentiation. Moreover, no association was observed between the age, sex of patients or location of the carcinoma and smoking duration.

The ROC curve analyses revealed that three miRNAs (miR-31, LET-7a, miR-33) can effectively discriminate between patients with LSCC and healthy controls however, no significant differences between these three miRNA serum levels became apparent with the clinicopathological features of the disease using Spearman correlation test Table 4.

**Table 4:** Correlation of serum levels miR-31, LET-7a, miR-33 with clinical characteristics

Serum		Age	Gender	Pack-years smoking	Tumor grade	Tumor location	Nodal involvement (N)	Primary tumor size (T)	Clinical stage	Treatment
miR-31	**r**_**s**_	**−0,06**	**− 0,17**	**− 0,11**	**− 0,03**	**− 0,06**	**0,04**	**− 0,07**	**−0,02**	**0,14**
	p	0,58	0,17	0,40	0,82	0,65	0,76	0,60	0,85	0,25
LET-7a	**r**_**s**_	**−0,03**	**−0,10**	**0,08**	**−0,13**	**− 0,09**	**−0,11**	**− 0,02**	**0,04**	**0,08**
	p	0,84	0,43	0,55	0,30	0,47	0,36	0,85	0,78	0,51
miR-33	**r**_**s**_	**−0,09**	**−0,17**	**0,03**	**0,13**	**0,01**	**−0,01**	**−0,05**	**− 0,03**	**0,24**
	p	0,47	0,18	0,84	0,31	0,91	0,91	0,71	0,85	0,06

The Spearman’s rank correlation coefficient (r) and (p) is presented

## Discussion

The present study evaluates the potential of serum miRNAs as molecular neoplasm biomarkers for discriminating patients with laryngeal squamous cell carcinoma from healthy controls. It has already been noted that plasma and serum samples are a serviceable blood-based source for epigenetic and genetic analysis.

The panel of miRNAs selected for the study was differently expressed (*p* < 0.05) in the serum of LSCC patients and healthy controls. The miRNAs displaying the greatest differentiation between groups were validated by RT-qPCR in the cohort. Eight miRNAs were found to be significantly up-regulated and three significantly down-regulated. Of these eleven, three serum miRNAs strongly differentiated healthy individuals from LSCC patients and could serve as valuable high accuracy biomarkers.

No correlation was found between serum expression of miRNAs, tumor size, disease stage and lymph node metastasis; however, Zhang et al. [11] report that miR-23 overexpression significantly correlated with clinical stage of the disease and five-year survival rate (p,0.01). Also, Arantes et al. [12] note enhanced expression of miR-21 in patients with head and neck squamous cell carcinoma (HNSCC), resulting in poor response to chemoradiation and worse survival rate. Single reports have even found that some miRNAs, such as miR-221 and miR-378, could serve as molecular neoplastic biomarkers for determining the effect of surgical treatment in patients with laryngeal carcinoma [13, 14].

Our previous study, based on samples from tumor tissues, like most diagnostic expression profiling of miRNAs, found miR-885-5p and PIK3R to be the best indicators for the classification of laryngeal cancer tissue and normal mucosa [15]. This and several other studies have reveal the diagnostic and prognostic value of circulating miRNAs from serum and plasma regarding laryngeal cancer [14, 16, 17].

Few studies have so far examined the value of serum miRNA expression for diagnosing laryngeal cancer. Our present study found eight miRNAs to be up-regulated in LSCC patients (miR-31, miR-141, miR-149a, miR-182, miR-485-3p, miR-122, miR-33 and LET-7a). Although no report has so far confirmed the role of one of them, miRNA-31, in the serum of LSCC patients, overexpression of miRNA-31 in esophageal SCC (ESCC) in serum and cancer tissue has been associated with poorer prognosis in relapse-free survival and tumor-specific survival, metastasis [11] and enhanced radiosensitivity [18]. The role of up-regulated miRNA-182 in our patients has also been confirmed in an earlier in vitro study on cancer cell lines which found overexpression of miRNA-182 to be closely associated with p53 overexpression, and to promote cell proliferation and migration in HNSCC [19]. miR-145 was also found to be down-regulated in our LSCC patient group; it has also been found to act as a tumor suppressor in patients with esophageal SCC [20]. However, an in vitro study on an adenocarcinoma esophageal cell line by Derouet et al. [21] revealed that, contrary to the squamous cell line, elevated miR-145 expression enhanced cell invasion and facilitated distant metastasis by anoikis protection.

Besides miR-145, only two other miRNAs, miR-133a and miR-223, were down-regulated in the serum of our patients. A similar miRNA signature, with lowered miR-133a, miR-133b and miR-145 expression, was found in subjects with ESCC [22]. Furthermore, Kano et al. [22] report that these miRNAs directly control oncogene FSCN1 (Fascin Hounds 1 gene), which takes part in the carcinogenesis of ESCC. The role of miR-223 remains controversial. While Xi Zeng et al. report that miR-223 expression was reduced in the serum of nasopharyngeal carcinoma patients compared to non-cancerous individuals [23], another study has found plasma miR-223 levels to be significantly higher in patients with pancreatic neoplasm compared to healthy controls [24]. It is possible that this miRNA plays a variable role as either oncogene and suppressor in different tumors. However, no other studies have examined the relationship between these types of miRNA in serum LSCC.

Members of the Let-7 miRNA family have been reported to be differentially expressed in various types of cancers [25], and serum miRNA Let-7a was found to be overexpressed in LSCC subjects in the present study. In contrast, Song et al. [26] demonstrated significant down-regulation of Let-7a in laryngeal cancer tissue compared to adjacent normal tissue, including 59 paired laryngeal tissues, and that Let-7a expression negatively correlated with higher TNM stage and lymph node metastasis. Such conflicting data may be due to their choice of specimens, i.e. tissue rather than serum, or may be related to different world regions with their unique environmental factors or population characteristics [27]. Even smoking habit and alcohol consumption can significantly alter miRNA expression [28].

In addition, ROC curve analysis of the signature of eleven serum miRNAs identified three which perfectly discriminated healthy individuals and LSCC patients: miR-31, miR-33 and Let-7a. Their accuracy for identifying LSCC was 99, 100 and 99%, respectively, with sensitivity and specificity ranging from 98 to 100%. However, the significance of the present results should be validated in patients with early stage of the disease to provide data about their value as early molecular neoplastic biomarkers.

The miRNAs in the blood and another human fluids are have great value as potential biomarkers due to their high stablity in biological samples and strong resistance to exogenous and endogenous RN-ases. Furthermore, they are more readily sampled than tissue miRNAs [29]. Therefore, novel biomarkers are immediately required for the enhancement of diagnostic, prognostic and therapeutic appliance.

Our findings indicate that eight of the studied miRNAs were significantly expressed in the serum of LSCC patients compared to healthy controls, and that three of them, viz. miRNA-31, miRNA-33 and Let-7a, strongly discriminated healthy individuals from LSCC patients. This clearly indicates the additive effect of these three miRNAs with regard to their diagnostic value: their expression in serum can be used to diagnose LSCC with patients with high sensitivity and specificity.

Hence, the levels of circulating miRNAs in serum may serve as promising potential diagnostic biomarkers for patients with laryngeal cancer. This study provides further evidence that the levels of individual miRNAs or the miRNA profile may be useful as serum biomarkers in patients with laryngeal cancer; however, further investigations are needed to validate their potential applicability in the early diagnosis of laryngeal human cancer and their prognostic value. Nevertheless, our findings indicate that miR-31, LET-7a and miR-33 may serve as novel non-invasive biomarkers for LSCC.

## Conclusion

The expression levels of miR-31, miR-141, miR-149a, miR-182, LET-7a, miR-4853p, miR-122, miR-33 in serum were up-regulated in pre-operative LSCC patients compared to healthy controls, while the expression levels of miR-145, miR-223 and miR-133a were significantly down-regulated. Of these, three miRNAs strongly discriminated healthy controls from LSCC patients: miRNA-31, miRNA-33 and Let-7a.

## Abbreviations

AJCC: American Joint Committee on Cancer
AUC: Areas under the ROC curve
ESCC: Esophageal squamous cell carcinoma
H&E: Haemotoxylin and Eosin
HNSCC: Head and neck squamous cell carcinoma
HPV: Human papillomavirus
LC: Laryngeal cancer
LSCC: Laryngeal squamous cell carcinoma
mRNAs: Human messenger RNAs
ROC: Receiver-operator characteristic
RT-qPCR: Real-time quantitative polymerase chain reaction
UICC: Union for International Cancer Control
